# Optimisation of a standalone photovoltaic electric vehicle charging station using the loss of power supply probability

**DOI:** 10.1016/j.heliyon.2023.e20836

**Published:** 2023-10-10

**Authors:** Zhendong Chen, Aritra Ghosh, Neil Stephen A. Lopez

**Affiliations:** a Faculty of Environment, Science and Economy (ESE), Renewable Energy, Electric and Electronic Engineering, University of Exeter, Penryn TR10 9FE, UK; b Department of Mechanical Engineering, De La Salle University, Manila, 0922, Philippines

**Keywords:** Electric vehicles, Photovoltaics, Charging station, Loss of power supply probability (LPSP)

## Abstract

The UK is planning to ban the sale of fuel vehicles entirely by 2035 and electric vehicles will be a potential alternative to fuel vehicles. The increase in electric vehicles will increase the charging demand. Standalone charging stations are a potential solution to alleviate the grid challenges of increased charging demand. In this work, the authors investigate a reliability analysis of a 2 MW standalone photovoltaic electric vehicle charging station (PVEVCS) using the loss of power supply probability(LPSP). The PVEVCS model consists of a PV system, a battery energy storage system (BESS) and a CS, using the climate data from Camborne, UK and classifying it into high and low irradiation sections. Next, four different charging demand profiles are selected to examine the models’ LPSP. Later, the chosen charging demand profiles are optimised using various combinations of PV systems, BESS and CS. It is concluded that the different solar irradiation had a significant effect on the LPSP. Under the same combination, higher PV capacity has a more positive impact on reducing daytime LPSP, higher BESS capacity has a more significant effect on lowering nighttime LPSP and larger CS capacity has a more significant impact on declining hourly LPSP.

## 1 Introduction

The transport sector consumes enormous amounts of oil and gas resources and produces significant carbon emissions, which have harsh environmental impacts [[1], [2], [3], [4]]. One hundred ninety-seven countries signed the Paris Agreement in 2015 to suppress rising global temperatures by reducing carbon emissions [5]. To reduce carbon emissions from the transport sector, the UK government announced a ban on the sale of fuel cars by 2030 and zero carbon emissions from the vehicle sector by 2035 [6]. Electric vehicles(EVs) are considered one of the best alternatives to fuel vehicles because of their zero tailpipe emissions, which can potentially reduce greenhouse gas emissions by up to 90 % [7,8]. In Europe, new registrations of electric vehicles reached 1.4 million in 2020, while in the UK, new electric vehicle registrations reached 176,000, twice as many as in 2019 [9].

The increase in EVs presents two potential challenges. (1) One is that charging EVs can cause instability in the grid. The increase in the number of electric vehicles will cause an increase in the demand for electricity, which typically comes from the distribution grid [10]. A large number of EVs charging can cause harmonic distortion and disturb voltage stability; particularly, the grid becomes increasingly unstable as the increased charging demand for EVs [11,12]. Once EVs become the dominant mode of transport in the UK, ensuring the load capacity and stability of the grid will be crucial. (2) The other is that EVs are questionable for reducing carbon emissions. From a life cycle assessment perspective, the CO2 emissions of EVs are strongly influenced by the source of electricity, the more electricity is generated from renewable sources (wind, hydro, solar, etc.), the lower the carbon emissions of EVs [13,14]. Therefore, to reduce carbon emissions in the transport sector more efficiently, developing EVs and increasing the share of renewable energy in the transport sector is necessary.

To address these challenges, charging stations (CS) with integrated stand-alone photovoltaic(PV) power systems, also called photovoltaic electric vehicle charging stations(PVEVCS), are widely considered [[15], [16], [17]]. This will not be connected to any grid and generates electricity solely from PV power systems [18]. It usually consists of three components, the PV generation system, the BESS and the CS; when the PV power generation is higher than the electricity demand, it charges the BESS to store electricity, while when the PV generation is lower than the electricity demand, the BESS charges the EVs [19,20]. In particular, as it does not require a connection to the grid, it can also be built in areas where the electricity system is not well developed [21,22]. A self-shading parallel reflector-based bi-facial solar PV technology was designed and considered for was intended to be considered for application in standalone PVEVCS in Leeds, UK [23].

Previously, a great deal of research has been done on PVEVCS. Colak et al. ([24] constructed a model to predict the total output of PVEVCS. They simulated it using weather data from Ankara city, Turkey (39°55′31.9188″N, 32°51′58.6332″E), which is feasible, efficient and cost-effective under the given conditions. Ribeiro et al. [25] produced a Multi-Criteria Decision Analysis tool to assess the prospects of the power generation sector, covering three dimensions: technical, economic and policy. The device can also evaluate the suitability of the location for stand-alone PV systems. Sara and Vincent used the characteristics of the SPEIC converter to design a PVEVCS that can provide constant voltage charging to the BESS, even if the output voltage of the PV is over a wide range [26]. Caines et al. evaluated the impact of BESS on the independence of a grid-connected PV charging station, found that the effectiveness of BESS in a grid-connected PV charging station is strongly influenced by the pattern of charging demand profile [27]. Singh et al. assessed the feasibility of a standalone PV charging station in India, resulting that although the land utilisation of a standalone charging station is 16 times more than a grid-connected charging station, it is more advantageous in remote areas and areas where the grid is not well developed [28]. Akhtar team optimised the capacity of BESS in terms of reducing peak loads, minimising BESS costs and enhancing EVs' resilience [29]. Kádár and Varga [30] studied the charging combinations of different PV-EV. The study showed that the parking area in the workplace during the day is a feasible charging area. They propose a delayed charging strategy to moderate charging peaks. Satya Prakash Oruganti et al. [31] presented a design of a small-capacity and mobile PVEVCS for situations where EVs need to be charged urgently but cannot access a charging station. In addition, studies have shown that in standalone systems, energy losses can be reduced by up to 10 % due to the avoidance of DC-AC conversions, thus improving energy efficiency [32,33]. Dai et al. [34] used the Multi-Agent Particle Swarm Optimisation Algorithm to optimise the capacity of the PV system and battery energy storage system (BESS) and to optimise the battery charging and discharge patterns to improve energy efficiency. Zhao and Burke [35] optimised the energy management of the CS by considering PV power forecasts and charging demand (CD) to maximise the use of PV power for charging electric vehicles. In addition to the single PV system, some designs incorporate other renewable energy generation systems, such as wind energy conversion systems [36,37], hydropower [38,39] and biogas [40]. Zieba Falama et al. [41] LPSP and energy cost as objective functions for optimising a standalone PV system with BESS in remote areas. Bilal et al. [42] developed a technical-economic and environmental analysis of a grid-connected EVCS with hybrid energy systems, using LPSP, total net present cost and levelized cost of energy cost as objective functions. Ghanjati and Tnani [43] optimised the sizing and energy management of a standalone hybrid system with standalone PV, battery and pumped storage systems using a Genetic Algorithm that considered LPSP and levelized cost of energy as objective functions.

Literature commonly focuses on designing hybrid energy systems integrated with PVEVCS or concerns the balance between the supply side of the charging station and the charging demand. It is evident that there is a considerable gap in assessing the stability of a standalone EVCS, especially the impact of multiple factors. The main object of this paper was to assess the stability of a standalone PVEVCS using LPSP. The factors considered were PV capacity, BESS capacity and CS capacity. Four charging demand profiles have been adopted for the variability. Four charging files were considered for four charging scenarios and the capacities of charging stations were optimised based on these scenarios.

## 2 Materials and methods

This section meticulously encapsulates the analytical framework of this study. It begins by outlining the simulation approach before the design of the whole systems. The indicator for evaluating the reliability of the system, LPSP, is subsequently appraised. Finally, the assumptions underlying the entire research are stated.

### 2.1 Simulation approach

**Fig. 1:**
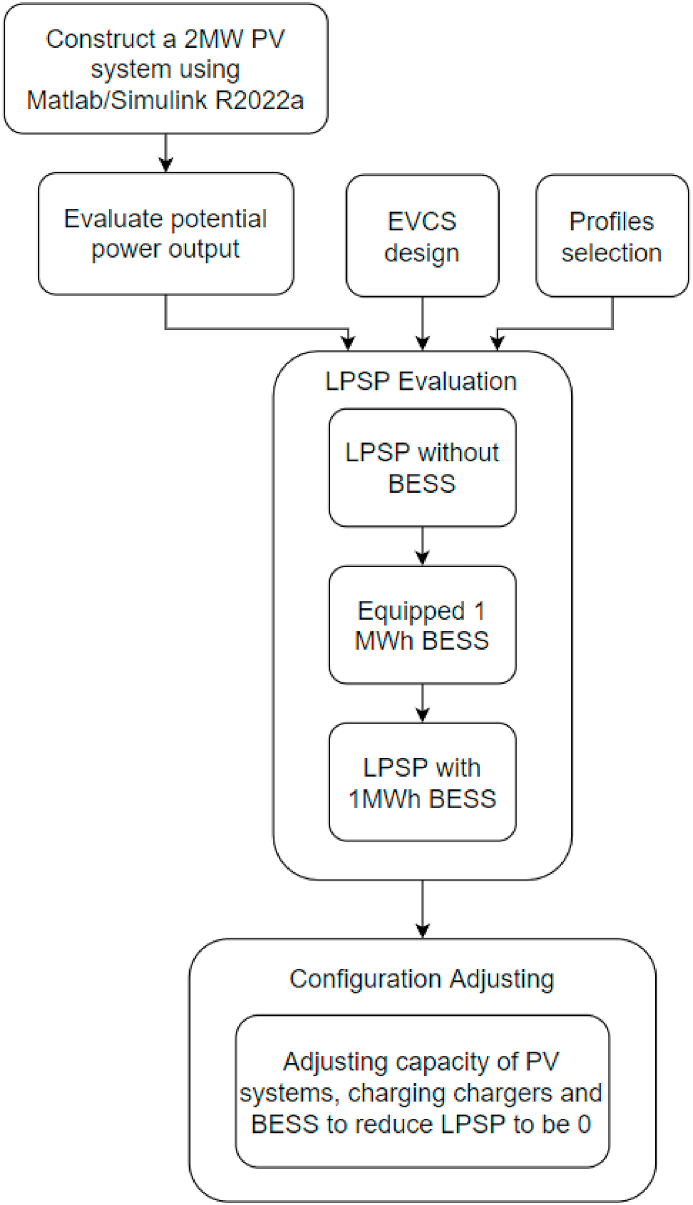
Modeling approach flow chart.

Fig. 1 presents the flow chart for this work. Firstly, constructing a standalone 2 MW PV system exclusive BESS for EVCS applying Simulink R2022a. Subsequently, evaluating the potential power output using specific solar resources, designing an EVCS and selecting four charging demand profiles additionally. Following this, evaluate the LPSP of four profiles under EVCS without BESS. Thereafter, equipped with 1 MWh BESS to investigate the LPSP change. Finally, using various capacities of PY systems, charging chargers and BESS to reduce LPSP to 0.

**Fig. 2:**
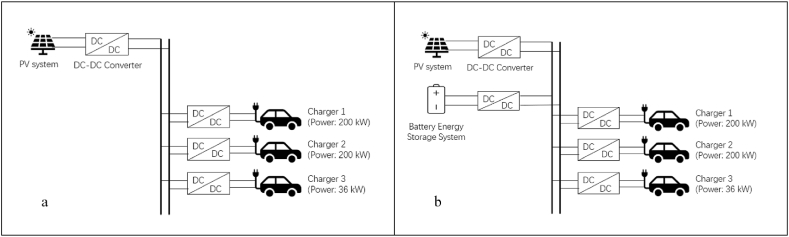
The framework of Photovoltaics electric vehicle charging stations. (a) Framework without a battery storage system. (b) Framework with a battery storage system.

The framework of PVEVCS contains three parts, a PV system, a battery storage system and a charging station. Fig. 2 (a) presents the structure of PVEVCS without BESS, while Fig. 2 (b) displays the form of PVEVCS with BESS. They are standalone systems powered only by the PV system, which converts solar energy into electricity and supplies it to the CS and BESS. All components are connected to the DC bus via a DC/DC converter.

In the morning, the overall power relationship was calculated in Equation (1):

(1) $$P=P_{c}+P_{b}+P_{w}$$

At night, the PV output is 0, so the energy relationship was calculated in Equation (2):

(2) $$P_{c}=P_{b}$$

Where P is the PV system power output, P_c_ is the power output of CS, P_b_ is the battery charging power and P_w_ is the daily wasted power.

For model 1, there is no BESS, so P_b_ is 0 in Equations (1), (2).

In this system，energy balance at EV charger side can be calculated by Equation (3:

(3) $$E_{charger}=E_{PV}\cdot \eta_{DCP}\cdot \eta_{TL}\cdot \eta_{DCC}\cdot \eta_{c}+E_{B}\cdot \eta_{DCB}\cdot \eta_{TL}\cdot \eta_{DCC}\cdot \eta_{c}$$

Where E_charger_ is the energy exported to EV, Epv is energy exported by PV systems, $\eta_{DCP}$ is the efficiency of DC converter of on PV systems side, $\eta_{TL}$ is the efficiency of transmission lines, $\eta_{DCC}$ is the efficiency of DC converters on the charging station sides, $\eta_{c}$ is the efficiency of EV chargers, EB is the energy output of BESS, $\eta_{DCB}$ is the efficiency of DC converters on BESS sides.

### 2.2 An overview of the PV system

**Fig. 3:**
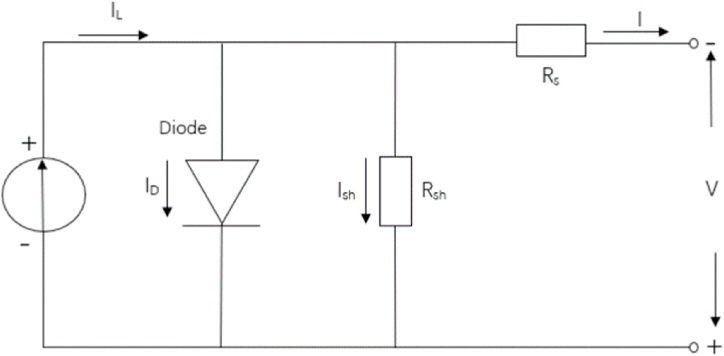
The equivalent circuit of PV modules [44].

The PV system equips the maximum power point tracking, the incremental conductivity method, in which the nominal power is 2 MW. The equivalent circuit for the PV modules is shown in Fig. 3.

Equations (4)–(7) [44] show the power output of the PV systems corresponding to Fig. 3.

(4) ID=I0[exp(VD/VT)-1]

(5) VT=kT/qnINcell

(6) I=IL–ID–Ish

(7) Ppv=IV

Where I_D_ is the diode current, I_0_ is the saturated diode current, V_D_ is the diode voltage, V_T_ is the diode voltage at specific temperatures, k is the Boltzman constant, q is the electron charge, T is temperature, nI is the diode ideality factor, N_cel_l is the number of cells connected in series, I is the PV current, V is the PV voltage and P is the PV output.

The energy generated by PV systems can be calculated by Equation (8)

(8) $$E_{pv}=n\;P_{pv}t$$

Where n is the number of PV modules utilised in PV systems, t is the time.

**Fig. 4:**
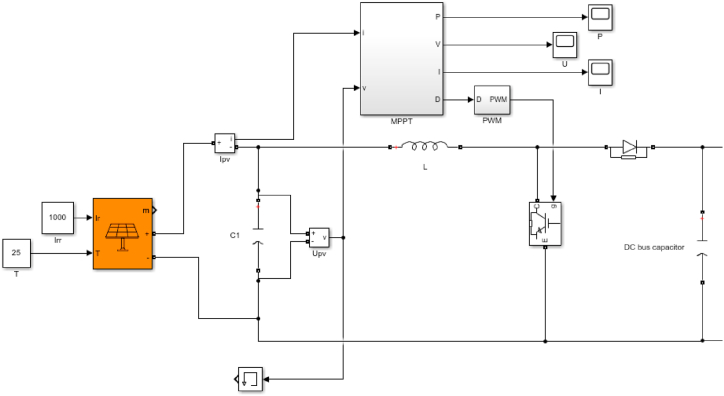
Schematic design of the 2 MW PV system.

**Fig. 5:**
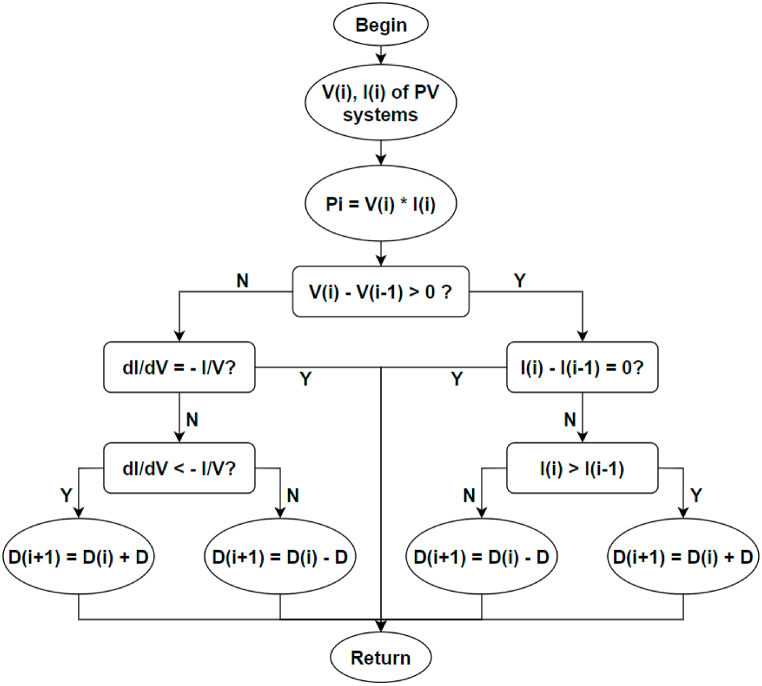
MPPT of incremental conductance method [45].

Fig. 4 illustrates the PV system schematic editing. A maximum power point tracking(MPPT) controller was utilised in the PV system to obtain the maximum power output. The applied MPPT is an incremental conductance method and its flow chart is shown in Fig. 5 [45].

The selected PV module details the for Simulink R2022a database [44], as shown in Table 1.

**Table 1:** PV module parameters.

Module	A10Green Technology A10J-M60-220
Maximum Power	219.876
Cells per module	60
Open circuit voltage V_oc_ (V)	36.06
Short circuit current I_sc_ (A)	7.95
Voltage at maximum power point (V)	30.12
Current at maximum power point (A)	7.3
Temperature coefficient of V_oc_ (%/deg.C)	−0.3624
Temperature coefficient of I_sc_ (%/deg.C)	−0.054805

The solar irradiation data was collected from Meteonorm 8.1, shown in Table 2. As Table 2 presents, there is a significant difference between several months. Therefore, using 100 kWh/m^2^ as the dividing line, 12 months were classified into two intervals: the low irradiation section (LIS), which contains the months that Ig is below 100 kWh/m^2^, while the high irradiation section (HIS), including the months that Ig is higher than 100 kWh/m^2^.

**Table 2:** The selected solar radiation data in Camborne, the United Kingdom.

Low Irradiation section		High Irradiation section	
Month	Ig	Month	Ig
January	26	April	127
February	43	May	158
March	82	June	168
October	58	July	157
November	30	August	136
December	21	September	102
Location: Camborne, UK; 50.2170°N, −5.3170°E			
Ig: Irradiation of global radiation horizontal (kWh/m^2^)			

**Fig. 6:**
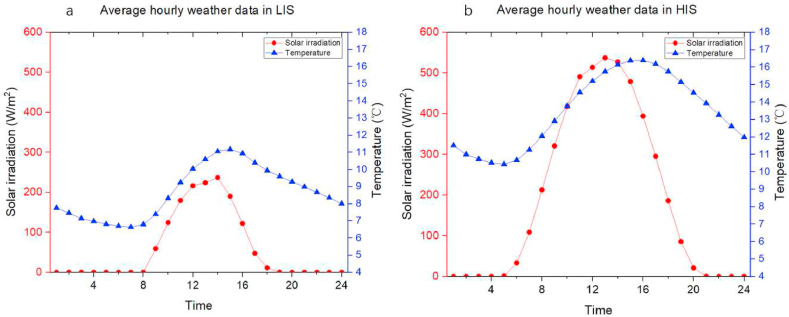
The average hourly weather data in two solar irradiation sections. (a) Data in the low irradiation section. (b) Data in the high irradiation section.

Then calculate the average hourly irradiation and temperature, shown in Fig. 6(a and b).

**Fig. 7:**
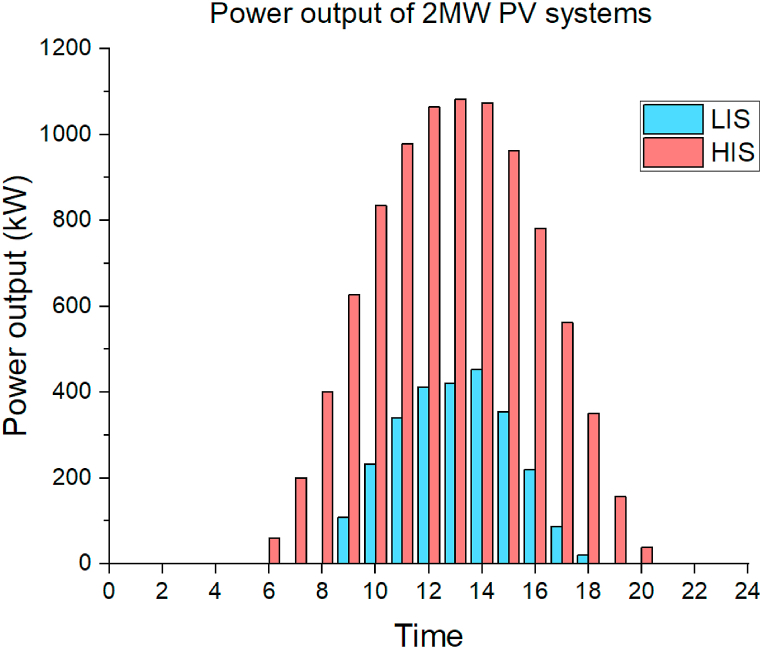
The hourly PV power generation of the 2 MW PV system in two irradiation sections.

The hourly irradiation and temperature data are the input parameters for the PV system to obtain the power output. The PV power output in LIS and HIS is shown in Fig. 7.

### 2.3 An overview of the charging station

Three chargers containing two 200 kW rapid-charging chargers and one 36 kW fast-charging charger, were initially considered for CS. Therefore, the maximum power output (P_cm_) can be up to 436 kW per hour and 10464 (436 kW*24 h)kWh per day. The information on chargers is shown in Table 3.

**Table 3:** The setting of the charging station.

Electric Vehicles Charging Station				
Parameter	Unit	Charger 1	Charger 2	Charger 3
Maximum voltage	V	1000	1000	450
Maximum current	A	200	200	80
Maximum Charging power	kW	200	200	36

### 2.4 An overview of the battery storage system

In this simulation, sealed lead-acid batteries were considered the battery storage system as it is low cost and maintenance-free [46]. The applied battery's parameters are presented in Table 4.

(9) $$E_{B}=E_{0}+\int_{0}^{t}V_{B}I_{B}dt$$

Where E_0_ is the initial energy in BESS, V_B_ is the BESS voltage of charging or discharging, I_B_ is the BESS current charging or discharging.

**Table 4:** Specifications of battery storage systems.

Battery Storage Systems Parameters	
Technology	sealed lead-acid
Battery type	AcmeG 12V 200
Nominal voltage	12.0 V
Capacity at C10	200 Ah
Static lifetime at 20 °C	12 years
SOC max	90 %
SOC min	20 %

Energy in BESS can be calculated by Equation (9) [47].

The state of charge(SOC) can be defined by Equation (10) [48]:

(10) $$\frac{\text{SOC}\left(t\right)}{\text{SOC}\left(t-1\right)}=\int_{T-1}^{T}\frac{P_{B}\left(t\right)\eta_{B}}{V_{B}}dt$$

Where P_B_ is the charging or discharging power, $\eta_{B}$ is the efficiency of charging or discharging.

In this simulation, the capacity of BESS was initially set to 1000 kWh. The state of charge of BESS was set as 20 %–90 %, which means that when the battery is below 20 %, it will stop discharging and when it is above 90 %, it will stop charging. The unused power generated by the PV system is stored in BESS and when the power from the PV system cannot cover the charging demand, the BESS will provide a part of the power. At the beginning of the simulation, the initial energy was set to 90 %.

### 2.5 The overview of charging demand profiles

Four charging demand profiles were selected for this simulation from published academic articles representing the daily charging demand.

**Fig. 8:**
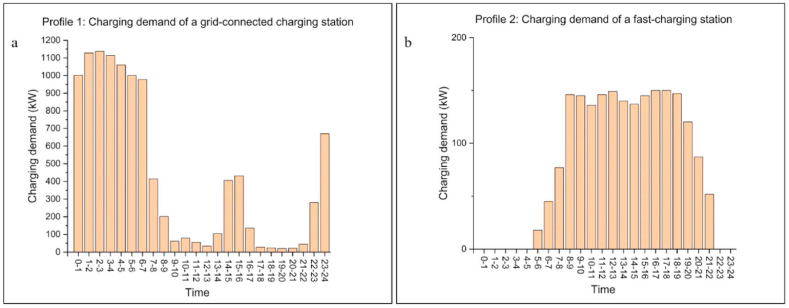
Charging demand profiles of Profile 1 and Profile 2. (a)A grid-connected CS (considered as Profile 1 in this work); Location: Zhengzhou, China; 34° 44′ 47.8068″ N, 113° 37′ 31.1808″ E; Taken from Tao et al. (2020). (b)A fast-charging CS (considered as Profile 2 in this work); Location: Italy; 42.76698° N, 12.493823° E; Taken from Mancini et al.(2020).

Profile 1 is from Tao et al. [49]. Tao simulated the daily charging demand of a grid-connected public charging station in Zhengzhou, China, using actual driving data from 1000 private cars over one year, shown in Fig. 8(a). Modelled data shows that charging demand is mainly concentrated in the latter part of the night from 23:00 to 7:00, while daytime demand is insignificant. The daily charging demand is 10433 kWh.

Profile 2 is from Mancini et al. [50]. Mancini forecasted the charging demand for a 150 kW fast-charging station, located in a commercial centre in Italy, as shown in Fig. 8(b). The daily charging demand is 1990 kWh.

**Fig. 9:**
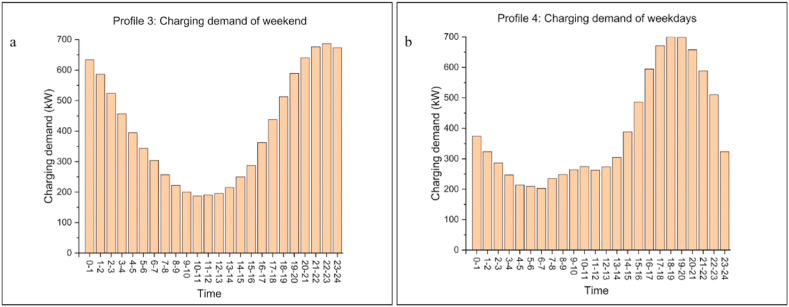
Charging demand profiles of Profile 3 and Profile 4. Location: Manila, Philippines; 14.582259° N, 120.9748° E; Taken from Lopez et al. (2021). (a) Charging demand of residential areas on the weekend (considered as Profile 3 in this work); (b) Charging demand of residential areas on the weekdays (considered as Profile 4 in this work).

Profiles 3 and 4 are from Lopez et al. [51], who used Discrete Event Simulation, embedded with Monte Carlo methods, to predict charging demand in residential areas in Manila, the Philippines, based on 1000 EVs, as shown in Fig. 9(a and b). They found that charging loads on weekdays differed significantly from those on weekends. Profile 3 is the charging demand on the weekend and the daily total charging demand is 9825 kWh. Profile 4 is the charging demand on weekdays and the daily total charging demand is 9336 kWh. The difference in daily charging demand between Profile 3 and Profile 4 is insignificant, but the distribution is different. The peak demand period for Profile 3 is 17:00–4:00, while the peak demand period for Profile 4 occurs between 14:00 and 23:00.

### 2.6 Loss of power supply probability

Loss of power supply (LPS) is used to measure the gap between power demand and power supply, which only occurs when the energy supply (E_s_) is lower than the energy demand (E_D_) [52]. Loss of power supply probability (LPSP) is used to evaluate the probability that hybrid energy systems do not cover the energy supply, defined as 0 to 1 [53]. When LPSP is 0, the energy supply meets the energy demand, while when LPSP is 1, the energy supply does not meet the energy demand.

LPS and LPSP were calculated by Equations (11)–(13):

(11) Es(t)=Epv(t)+EB(t)–EBm

(12) LPS(t)=ED(t)–Es(t)

(13) LPSP(t)=Σ_(t−1)ˆTLPS(t)/Σ_(t−1)ˆTE_D(t)

Where E_Bm_ is the minimum stored energy.

Further, energy is the product of power and time, expressed as Equation (14):

(14) E=Pt

Take Equation (14) into Equations (11)–(13) and simplify to get equation (15) (16):

(15) $$\text{LPS}\left(t\right)=P_{D}\left(t\right)\;–\;\text{Ps}\left(t\right)$$

(16) LPSP(t)=Σ_(t−1)ˆTLPS(t)/Σ_(t−1)ˆTP_D(t)

After simplifying, LPSP has been converted from energy-related to power-related. This experiment calculated LPS and LPSP by Equations (15), (16).

### 2.7 Definitions and assumptions

This section will describe the definitions and assumptions associated with this simulation work.

(1) Daytime was defined as solar irradiation greater than 0; nighttime was defined as solar radiation equal to 0.
(2) A single hour of PV power generation and charging demand did not accumulate to the next time interval.
(3) It was assumed that no energy was lost during the simulation. System losses, including line transmission losses, battery charging and discharging process losses, energy losses during charging and discharging, were not considered.
(4) A series of abbreviations were utilised in the optimisation section. For example, PV2B1-436 indicates a 2 MW PV system with 1 MWh BESS and 436 kW CS capacity. Similar abbreviations follow this naming pattern.

## 3 Results and discussion

### 3.1 The results and discussion for the initial models

**Fig. 10:**
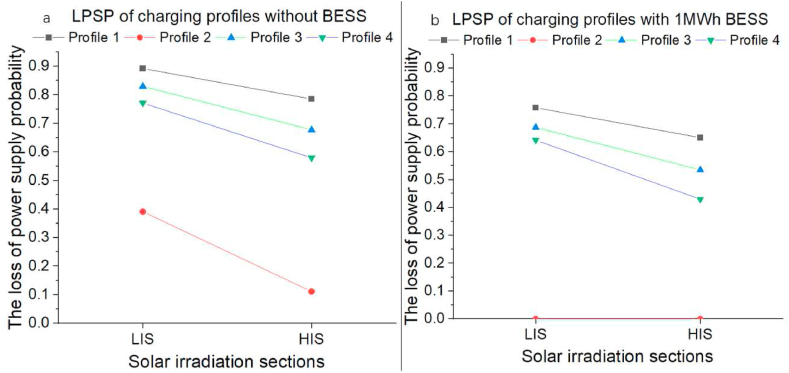
The LPSP for investigated charging demand profiles in two models. (a) The LPSP for PVEVCS without BESS. (b) The LPSP for PVEVCS with 1 MWh BESS.

Fig. 10(a) presents the LPSP results for charging demand profiles without BESS. In LIZ, the PV system produces low power generation due to low solar irradiation. Consequentially, the LPSP is very severe at LIS, even though profile 4 has a total energy requirement of only 1990kwh, and its LPSP is over 30 %. At HIS, the LPSP decreases due to the increase in PV power generation at high solar irradiation.

Fig. 10(b) displays the LPSP for four charging demand profiles in HIS with 1 kWh BESS. A part of the unused PV energy was stored in BESS and released at night. Compared to the model without BESS, all LPSP in the simulations with BESS were reduced. Especially for profile 2, its LPSP is reduced to 0 for LIS and HIS.

The above simulation results demonstrate BESS's effectiveness in reducing the LPSP of standalone PVEVCS. In the next step, different combinations were applied to the charging demand profiles to decrease the LPSP. It is worth mentioning that the LPSP of Profile 2 reached 0 for the 2 MW PV system, 1 MWh BESS and 436 kW CS capacity, which means that the energy supply can cover the charging demand of Profile 2. Therefore, it did not involve further optimisation analysis.

### 3.2 The results and discussion for profile 1 optimisation

**Fig. 11:**
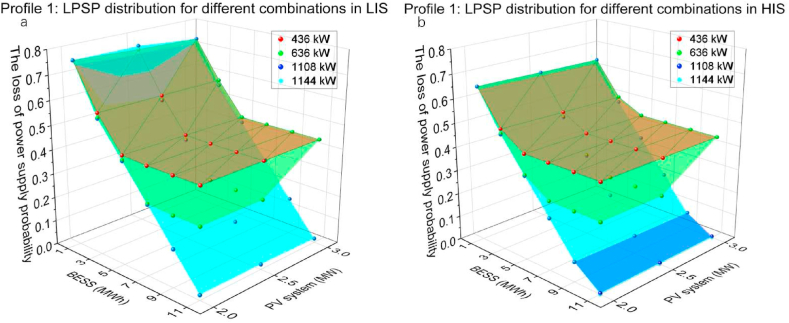
Profile 1: The LPSP of two irradiation sections. (a) The LPSP in LIS. (b)The LPSP in HIS.

The PV capacity of 2, 2.5, 3 MW and the battery capacity of 1, 3, 5, 7, 9, 11 MWh and the CS capacity of 436, 636, 1108, 1144 kW were utilised on profile 1. Fig. 11(a and b) presents LPSP of different combinations of PV systems capacities and BESS capacities, corresponding to CS capacity. Comparing two solar irradiation sections, the impact of solar irradiation on LPSP reduction is up to 14.2 %, with the combination of a 2 MW PV system and 1 MWh BESS. The increase in PV capacity does not effectively decrease LPSP, only up to 4 % of LPSP reduction. The charging peak of profile 1 is between 0 and 7 a.m. and the maximum charging demand is 1137 kW at 2–3 a.m. The starting time of the simulation is 0 a.m. so the critical factors for reducing LPSP for this file are the capacity of BESS and CS. With larger BESS capacities, more initial energy can be potentially consumed at the beginning of the simulation. The larger CS capacity, the limitation of power delivery will be reduced. The optimisation results indicate that the combination of 11 MWh BESS, 1144 kW CS capacity and 2 MW PV systems can decrease LPSP to 0 in HIS, while it reaches 0.0215 in LIS. With increasing PV capacity to 3 MW, the LPSP in LIS can be dropped to 0.0166.

**Fig. 12:**
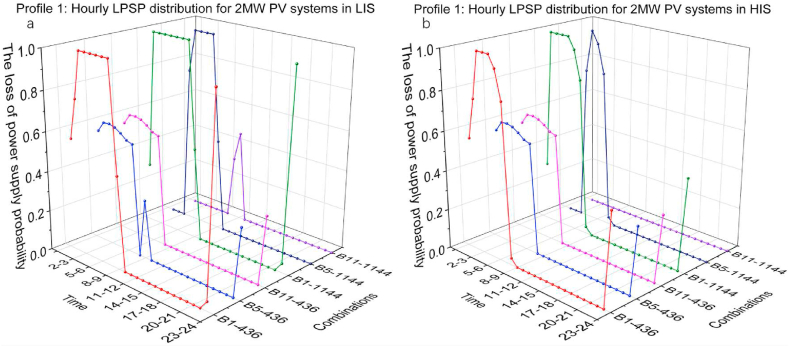
Profile 1: The hourly LPSP of a 2 MW PV system with different combinations. (a) The LPSP results in LIS. (b) The LPSP results in HIS.

**Fig. 13:**
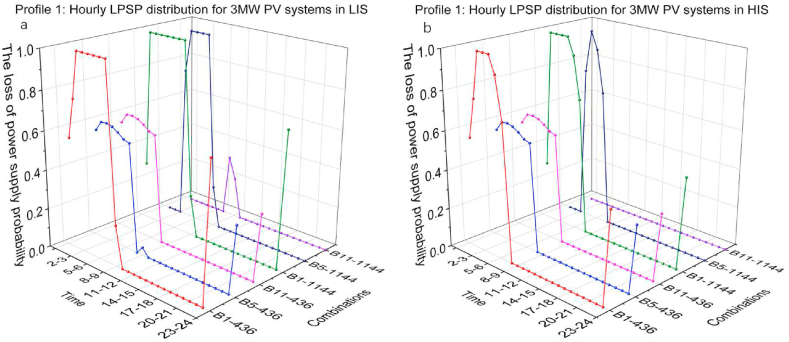
Profile 1: The hourly LPSP of a 3 MW PV system with different combinations. (a) The LPSP results in LIS. (b) The LPSP results in HIS.

Fig. 12(a and b) and Fig. 13(a and b) presents the hourly LPSP distribution of 2 MW and 3 MW PV systems capacity in LIS and HIS, which shows visually how the LPSP has changed. Label B1-436 means the combination of 1 MWh BESS and 436 kW CS capacity. As seen from this set of images, combinations of high CS and BESS capacity contributed to a dramatic reduction in LPSP in the first several hours. Comparing B5-436 and B11-436, although BESS capacity increased, changes of LPSP in first 7 h are the same, which illustrates that low CS capacity limits the energy output. Comparing B5-436 and B5-1144, LPSP from 0 to 1 a.m. in B5-1144 is much lower than it in B5-436, however, LPSP of B5-1144 in the following hours are higher than B5-4A larger CS capacity indicates that more energy is available to the EV charging demand in 1 h, also representing that the stored energy in BESS is consumed more rapidly. In contrast, lower CS capacity ensures that energy can be consumed for more time, although charging demand is only partially fulfilled. The charging demand in the daytime is much less than at nighttime, so the impact of increasing capacity of PV systems, BESS and CS on the reduction daytime LPSP is insignificant.

### 3.3 The results and discussion for profile 2 optimisation

LPSP of the initial combination, 2 MW PV systems, 1 MWh BESS and 436 kW CS, is already 0, but finding a smaller combination to meet 0 LPSP is meaningful in reducing cost, although the financial analysis was not an objective of this research.

**Fig. 14:**
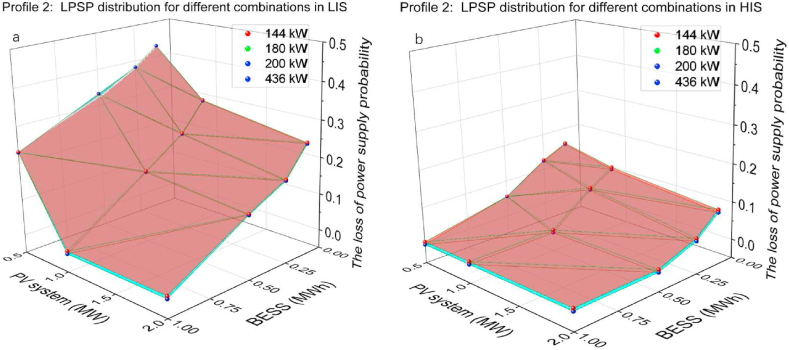
Profile 2: The LPSP of two irradiation sections. (a) The LPSP in LIS. (b)The LPSP in HIS.

The PV systems capacity of 0.5, 1, 2 MW, BESS capacity of 0.1, 0.25, 0.5, 1 MWh and CS capacity of 144, 180, 200, 436 kW were utilised to find the critical combination where LPSP is 0 and results are shown in Fig. 14(a and b). Starting with 1 MWh BESS and 2 MW PV systems, the critical combination was found by reducing the capacity of PY systems, BESS and CS. As the peak charging demand is 150 kW, 144 kW CS capacity, equal to four 36 kW chargers, could not cover entirely charging demand. Therefore, 180 kW CS capacity, equal to five 36 kW chargers, is the critical point that provides enough charging output. Combinations with 0.1 and 0.25 MWh BESS could not ensure LPSP to be 0 in LIS or HIS, whatever the capacity of PV systems and CS. In low PV systems capacity, especially in 0.5 MW, the impact of solar irradiation is meaningful, which results in 60 %–75 % LPSP reduction. In LIS, increasing BESS capacity has a more noticeable effect on LPSP than in HIS. Because of low solar irradiation, the power output of PV systems is limited and the large BESS capacity compensates well for the lack of PV systems output. In HIS, meanwhile, the power output of PV systems is sufficient to cover the charging demand and the increase in BESS capacity has a limited impact on reducing LPSP. The smaller combination to ensure LPSP is 0 in LIS and HIS is 1 MW PV systems, 0.5 MWh BESS and 180 kW CS.

### 3.4 The results and discussion for profile 3 optimisation

**Fig. 15:**
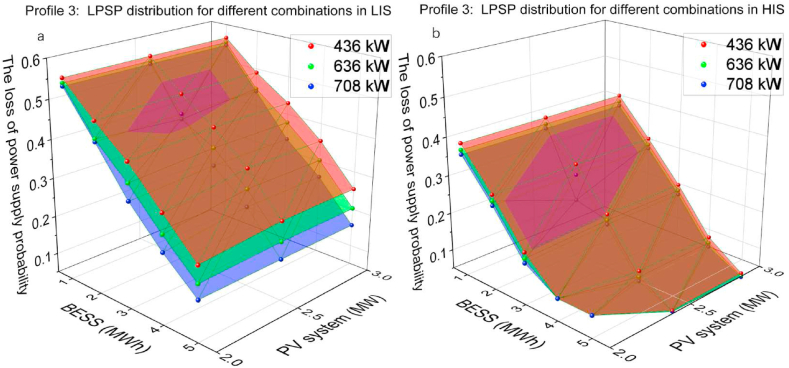
Profile 3: The LPSP of two irradiation sections. (a) The LPSP in LIS. (b)The LPSP in HIS.

The PV capacity of 2, 2.5, 3 MW and the BESS capacity of 1, 2, 3, 4, 5 MWh and the CS capacity of 436 kW, 636 kW and 708 kW were utilised on profile 3 for optimisation. Fig. 15(a and b) presents the simulated results for the optimisation of profile 3. Comparing the results in LIS and HIS, the impact of increasing solar irradiation on LPSP reduction is meaningful, with up to 100 % reduction from 0.157 drops to 0 with the combination of 3 MW PV systems, 5 MWh BESS and 708 kW CS. The increased PV capacity slightly affects LPSP with 1 and 2 MWh BESS in both solar irradiation sections, between 4 % and 10 % reduction. The effect of increased PV capacity starts to be significant in the 3 to 5 MWh BESS configuration, with up to 39 % LPSP reduction, from 0.254 drops to 0.157. Unlike the profile 1 results, the impact of solar irradiation on LPSP is more visible, which indicates that the solar irradiation is an essential factor for LPSP.

**Fig. 16:**
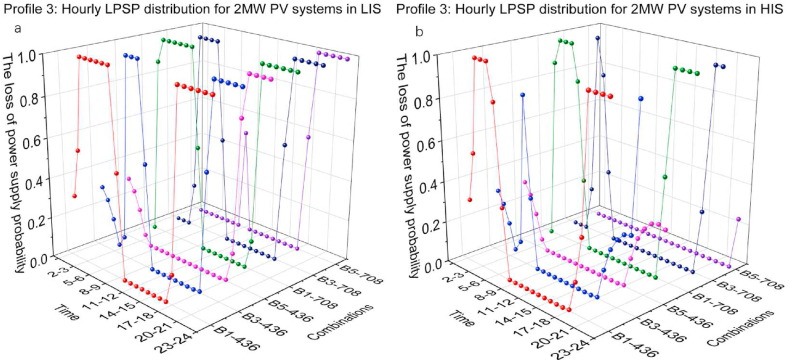
Profile 3: The hourly LPSP of a 2 MW PV system with different combinations. (a) The results in LIS. (b) The results in HIS.

**Fig. 17:**
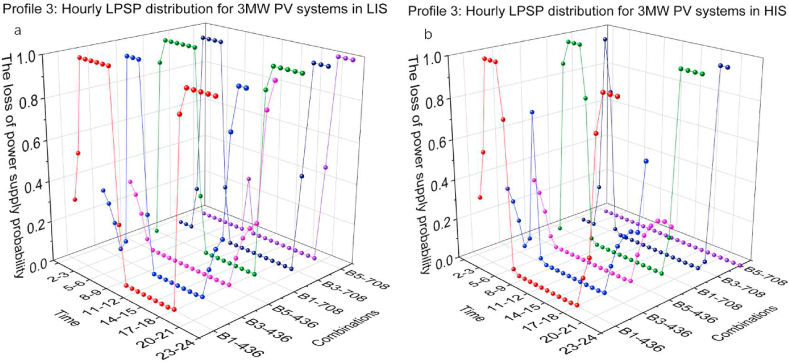
Profile 3: The hourly LPSP of a 3 MW PV system with different combinations. (a) The results in LIS. (b) The results in HIS.

Fig. 16(a and b) and Fig. 17(a and b) present the hourly LPSP of Profile 3 with different combinations of 2 MW and 3 MW PV capacity in LIS and HIS. Similar to the optimisation results of the simulations in profile 1, the high BESS capacity contributes significantly to charging demand relief at night. Comparing the nightly results of B3-436 and B5-436, 436 kW CS capacity was insufficient to cover the charging demand, although the energy in BESS was available. Comparing the LPSP distribution of 3 MW PV systems with B1-436 in LIS and HIS, the duration of nighttime in HIS is shorter than in LIS, resulting in the PV systems starting operation earlier so that the hourly LPSP drop is earlier in the HIS than in LIS. Simulation results for the other groups showed similar trends.

### 3.5 The results and discussion for profile 4 optimisation

**Fig. 18:**
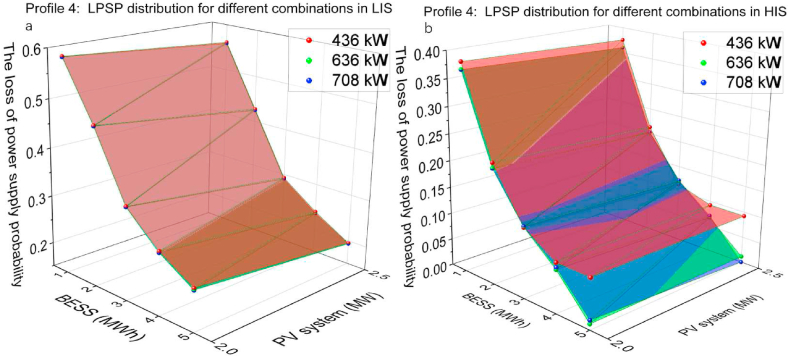
Profile 4: The LPSP of two irradiation sections. (a) The LPSP in LIS. (b)The LPSP in HIS.

The PV capacity of 2, 2.5 MW and the battery capacity of 1, 2, 3, 4, 5 MWh and the CS capacity of 436 kW, 636 kW and 708 kW were utilised on profile 4. Fig. 18(a) displays the simulated results for optimising profile 4 in LIS. It illustrates that the change in battery capacity has the most significant influence on LPSP. Under constant battery capacity conditions, the PV and CS capacity changes have not significantly impacted the changes to the LPSP. Fig. 18(b) illustrates the simulated outcome of profile 4 in HIS. Similar to the simulated results of profiles 1 and 3, the changes in PV capacity did not significantly impact LPSP. Nevertheless, the difference in CS capacity has had an appreciable effect on the LPSP. The combination that has the best performance is 2.5 MW PV systems, 5 MWh BESS and 708 kW CS, of which LPSP is 0 in HIS and 0.174 in LIS.

**Fig. 19:**
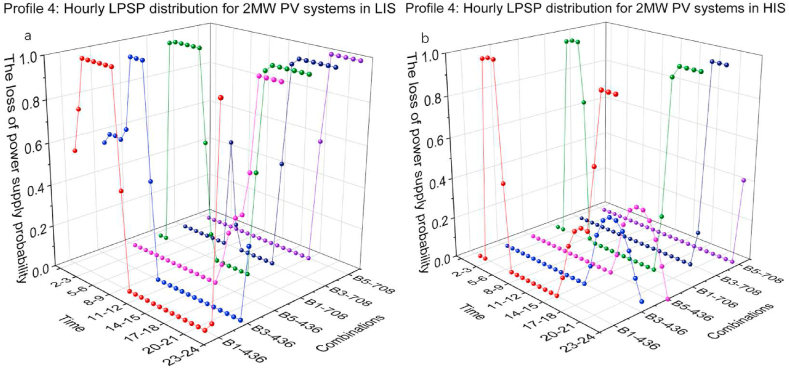
Profile 4: The hourly LPSP of a 2 MW PV system with different combinations. (a) The results in LIS. (b) The results in HIS.

**Fig. 20:**
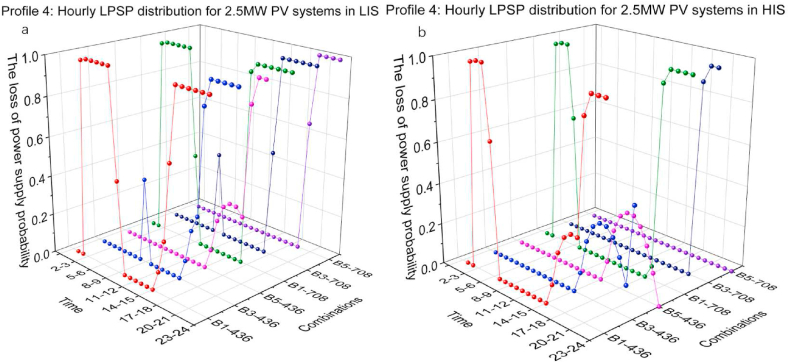
Profile 4: The hourly LPSP of a 2.5 MW PV system with different combinations. (a) The results in LIS. (b) The results in HIS.

Fig. 19(a and b) and Fig. 20(a b) present the hourly LPSP of Profile 4 with different combinations. At low BESS capacity, for instance, the set of B1-436, B1-708 for 2 MW and 2.5 MW PV systems, the LPSP on the night before sunrise varied sharply between 0 and 1. In LIS, because of the short daily duration, the LPSP is 1 for a longer time at night. In HIS, the PV power output can cover more of the charging demand due to the longer duration of sunlight and stronger solar irradiation.

## 4 Conclusion and future work

In this work, the LPSP of four charging demand profiles connecting a 2 MW PVEVCS without BESS was evaluated firstly for two solar irradiation sections, LIS and HIS. Later, various combinations of PV capacity, BESS capacity and CS capacity were utilised to optimise the LPSP.

The LPSP of the charging demand from grid-connected EVCS in Zhengzhou, China, Profile 1 in this work, is 0.892 for a 2 MW PV system, with 436 kW CS capacity but without BESS in LIS and 0.785 for 2 MW PVEVCS with 1 MWh BESS and 436 kW CS capacity. The combinations of 2, 2.5,3 MW PV systems, 1, 3, 5, 7, 9, 11 MWh BESS and 436, 636, 1108, 1144 kW CS capacity were utilised for optimising. After optimisation, the LPSP dropped to 0.016 in LIS for a combination of a 3 MW PV system, 11 MWh BESS and 1144 kW CS capacity, while in HIS, it dropped to 0 with the same combination.

The LPSP of the charging demand of Profile 2, which is from Italy, is 0.391 in LIS and 0.111 in HIS without equipping the BESS. After equipping a 1 MWh BESS, the LPSP dropped to 0 for LIS and HIS. Further, an optimisation analysis was done to find smaller combinations to ensure that the LPSP is 0. Results indicate that the combination of 1 MW PV systems, 0.5 MWh BESS and 180 kW CS is a better option for reducing the potential cost.

The LPSP of the charging demand of Profile 3, which is from the weekend in Manila, Philippines, is 0.830 in LIS and 0.677 in HIS for a 2 MW PV system with 436 kW CS capacity but without BESS. The combinations of 2, 2.5,3 MW PV systems, 1, 2, 3, 4, 5 MWh BESS, and 436, 636, 708 kW CS capacity were utilised for optimising. After optimisation, the LPSP decreased to 0.156 in LIS, equipping a 3 MW PV system, 5 MWh BESS and 708 kW CS capacity, while in HIS, the LPSP declined to 0 with the same combination.

The LPSP of the charging demand of Profile 4, the weekdays in Manila, Philippines, is 0.771 in LIS and 0.580 in HIS with a 2 MW PV system, no BESS and 436 kW CS capacity. With a 1 MWh BESS, it dropped to 0.718 in LIS and 0.625 in HIS. After optimisation, it decreased to 0.194 in LIS and 0 in HIS using a 2.5 MW PV system, 5 MWh BESS and 708 kW CS capacity.

In addition, PV capacity, BESS capacity, CS capacity and solar irradiation have the following impact on LPSP.

(1) High PV capacity contributes to the reduction of daytime LPSP.
(2) High BESS capacity effectively reduces LPSP at night.
(3) CS capacity only affects the LPSP if the charging demand is higher than CS capacity. If the PV power output can cover the charging demand and the charging demand does not exceed the CS capacity, increasing the CS capacity has no effect on the daily LPSP.
(4) CS capacity impacts the hourly LPSP at night, especially at low BESS capacity. At low BESS capacity, high CS capacity can contribute to a rapid depletion of stored energy, resulting in subsequent charging demand not being fulfilled.
(5) LPSP will differ for the same PV, CS, and CS capacity combination in different solar irradiation sections. In the low irradiation section, which considered months 1,2,3,10,11,12 in this work, the LPSP increased, while in the high solar irradiation section, which through months 4, 5, 6, 7, 8 and 9 in this work, the LPSP decreased.

In further works, the impact of long-time period charging demand on the LPSP of a standalone PVEVCS can be explored. Other potentially valuable extensions to this work are how limitations of EV charging speeds and financial factors constrain the standalone PVEVCS LPSP and multi -objective optimisation analysis that considers financial factors.

## Data availability statement

Data will be made available on request.

## CRediT authorship contribution statement

**Zhendong Chen:** Data curation, Formal analysis, Investigation, Methodology, Resources, Software, Validation, Visualization, Writing – original draftWriting – original draft, Writing – review & editingWriting – review & editing. **Aritra Ghosh:** Conceptualization, Formal analysis, Funding acquisition, Investigation, Methodology, Project administration, Resources, Software, Supervision, Validation, Visualization, Writing – original draftWriting – original draft, Writing – review & editingWriting – review & editing. **Neil Stephen A. Lopez:** Data curation.

## Declaration of competing interest

The authors declare that they have no known competing financial interests or personal relationships that could have appeared to influence the work reported in this paper.

## Nomenclature

BESS: Battery energy storage system
CS: Charging station
CS_M_: Maximum output of charging station
EV: Electric vehicle
HIS: High irradiation section
I: PV current
I_0_: Diode saturation current
I_D_: Diode voltage
I_g_: Irradiation of global radiation horizontal
I_ph_: Photogenerated current
k: Boltzman constant
LIS: Low irradiation section
LPS: Loss of power supply
LPSP: Loss of power supply probability
MPPT: Maximum power point tracking
N_cell_: Number of cells connected in series
nI: Diode ideality factor
P: Power output
P_b_: Charging or discharging power of the battery
P_c_: Power output of charging station
P_cm_: Maximum power output of charging station
P_D_: Charging demand
P_pv_: PV power output
PV: Photovoltaics
PVEVCS: Photovoltaic electric vehicle charging station
q: Electron charge
T: Temperatures
V: PV voltage
V_D_: Diode voltage
V_T_: Diode voltage at specific temperatures
